# Delay in Human Neutrophil Constitutive Apoptosis after Infection with *Klebsiella pneumoniae* Serotype K1

**DOI:** 10.3389/fcimb.2017.00087

**Published:** 2017-03-27

**Authors:** Chen-Hsiang Lee, Seng-Kee Chuah, Wei-Chen Tai, Chia-Chi Chang, Fang-Ju Chen

**Affiliations:** ^1^Division of Infectious Diseases, Department of Internal Medicine, Kaohsiung Chang Gung Memorial HospitalKaohsiung, Taiwan; ^2^Chang Gung University College of MedicineKaohsiung, Taiwan; ^3^Division of Gastroenterology, Department of Internal Medicine, Kaohsiung Chang Gung Memorial HospitalKaohsiung, Taiwan

**Keywords:** innate immunity, virulence, pathogenesis, metastatic infection, capsular polysaccharide

## Abstract

*Klebsiella pneumoniae* serotype K1 is a major cause of invasive syndrome defined by liver abscess with metastatic infections at other body sites. This culprit is known to be resistant to neutrophil phagocytosis and bactericidal activity. We hypothesized that *K. pneumoniae* serotype K1 might regulate neutrophil apoptosis and enhance the survival of the infected neutrophils that might serve as a vector for dissemination of the bacteria. Two serotypes of *K. pneumoniae*, KP-M1 isolated from a patient with liver abscess and DT-X (an acapsular mutant strain of KP-M1), were used to infect human neutrophils. The infected neutrophils were examined for their cytotoxicity, annexin V staining, proteins, DNA fragmentation, cytokine production, and viability that are involved in apoptosis. We found that KP-M1 was not destroyed and the ingested bacteria survived within neutrophils. While the uninfected neutrophils became apoptotic within 10 h, the neutrophils infected with KP-M1 could survive up to 24 h post infection. Constitutive apoptosis of KP-M1-infected neutrophils was significantly delayed compared to that of DT-X-infected or uninfected neutrophils (*p* < 0.01). KP-M1 modulated the anti-apoptotic effects by down-regulating the ratio of Bax to Bcl-2 and Mcl-1, and then delayed caspase-3 activation in the neutrophils, which was accompanied by inducing the anti-apoptotic cytokine, IL-8. These data suggest that *K. pneumoniae* serotype K1 can prolong the lifespan of infected neutrophils by delaying constitutive apoptosis within the first several hours of infection.

## Introduction

*Klebsiella pneumoniae* is the leading cause of pyogenic liver abscess (PLA) in worldwide (Ko et al., 2002; Lee et al., 2005). One of the major concerns for patients infected with *K. pneumoniae* is the development of PLA and other invasive syndromes (Fang et al., 2007; Siu et al., 2012). Though the majority of the reports of invasive syndromes are from East Asia over the past 3 decades (Ko et al., 2002), an increasing number of similar cases have recently been reported in Western countries (Lederman and Crum, 2005; Ye et al., 2016).

More than 77 capsular serotypes have been identified, and *K. pneumoniae* K1 serotype has consistently been found to be causative of the invasive syndromes throughout East Asia (Siu et al., 2012). With the use of the multilocus sequence typing, almost all K1 strains belong to ST23, which suggests a clonal expansion that has spread globally (Struve et al., 2015). While clinical studies on *K. pneumoniae* serotype K1 have postulated that the capsule polysaccharide is related to the development of invasive syndromes (Lee et al., 2014), investigations as to how it is capable of establishing an infection and spreading within the host cells are yet to be performed.

Polymorphonuclear leukocytes (PMNs) and serum complement are the two important components of innate immunity against the invading pathogens. In the cases of invasive syndromes caused by *K. pneumoniae*, the interactions between PMNs and *K. pneumoniae* serotype K1 have attracted research interest in that the PMNs may facilitate or impede the dissemination of *K. pneumoniae* (Lin et al., 2010). Apoptotic death in infected host cells may be caused by the bacterium as it proliferates and migrates from one cell to another (Kespichayawattana et al., 2000). PMNs undergo constitutive apoptosis upon leaving the bone marrow if pro-survival stimuli are lacking (Savill, 1997). To reduce the risk of disseminated infection, PMNs need to be healthy; it is of paramount importance to ensure their survival and control in order to prevent apoptosis (Savill, 1997).

In this study, we hypothesize that *K. pneumoniae* serotype K1 could spread by infecting circulating PMNs. We, therefore, used a multifaceted approach to test this hypothesis, and found that such an infection did not speed up PMNs death, but instead, lengthened cell lifespan through the impact on apoptotic pathways.

## Materials and methods

### Ethics statement

This study was carried out in accordance with a protocol approved by the Institutional Review Board of Chang Gung Memorial Hospital (Document no. 100-4029B). All subjects provided written informed consent in accordance with the Declaration of Helsinki.

### Isolation of neutrophils

Heparinized venous blood was obtained from 5 healthy adult volunteers. PMNs were isolated by dextran sedimentation followed by density gradient separation as described previously (Berends et al., 1994). Neutrophils were suspended in HBSS, counted, and diluted to 1.5 × 10^7^ cells/ml. The purity of each preparation was assessed by 0.25% trypan blue staining followed by a microscopic analysis. The suspensions contained 95–98% PMNs. Neutrophils were incubated at 37°C in a water bath in an Eppendorf tube with Ham's medium for further analyses. In all cases, replicate tests were performed using PMNs from different donors.

### Bacterial strains

KP-M1 (ST23; capsule serotype K1) was isolated from a patient with PLA. An acapsular *K. pneumoniae* mutant of DT-X was obtained by a subculture of strain DT-S (biotype *edwardsii*, capsule serotype K1, ST23; Lee et al., 2014). The lack of a capsule in DT-X was confirmed by staining with India ink. Bacteria were routinely cultured at 37°C in LB medium.

### Opsonization and infection of neutrophils

KP-M1 and DT-X were grown for 18 h in Trypticase soy broth (TSB, Difco, Lawrence, KS, USA), washed twice with a large volume of saline solution and adjusted to 1.5 × 10^7^ cells/mL in a Ham's medium. Bacteria were then opsonized (30 min, 37°C) in the presence of 5% fresh pooled human serum in Ham's medium and were added to the neutrophil cultures in pooled serum at a multiplicity of infection of 1 bacteria/neutrophil. Then, 0.3 mL of the mixed solution was incubated at 37°C for 30 min to allow infection to occur. This test was repeated five times with neutrophils isolated from five separate volunteers.

### Neutrophil bactericidal activity after infection with *K. pneumoniae*

Bactericidal activity was measured using a standard assay method (Hampton et al., 1994). After infection of PMNs as described above, samples (50 μL) were drawn at 0, 2, 4, 6, 12, and 24 h post-infection and diluted with 2.45 mL of H_2_O (pH 11.0) to lyse the neutrophils and sufficiently disperse the bacteria to permit evaluation by a colony assay. The plate was incubated overnight at 37°C and the number of colonies was counted. All tests were performed in triplicate to assure their reproducibility.

### Analysis of apoptotic cells with flow cytometry

PMNs were infected with *K. pneumoniae* (as above) or left untreated. At various time points post-infection, the apoptosis of PMNs was analyzed by flow cytometry using the FITC Annexin V Apoptosis Detection Kit (BD Biosciences, Franklin Lakes, NJ, USA) according to the manufacturer's instructions. Briefly, the cells were suspended in annexin V-binding buffer at a concentration of 10^6^ cells/mL. This suspension (100 μL) was stained with annexin V-FITC and propidium iodide. The cells were incubated for 15 min at room temperature. After adding 400 μL of binding buffer to each tube, the cells were analyzed by flow cytometry (FACSCalibur; BD Biosciences) using CellQuest Pro (Version 5.1).

### Cytotoxicity measurement using LDH assay

Culture supernatants were collected from PMNs infected with *K. pneumoniae* (see above) and untreated PMNs at various time points to determine for lactate dehydrogenase (LDH) activity. An LDH release assay was using a Cytotoxicity Detection Kit protocol (Clontech, Mountain View, CA, USA), following the manufacturer's protocol, with a few modifications. In brief, 200 μL of medium from each well was transferred to a new 96-well polystyrene plate and spun at 300 × *g* for 10 min. Next, 100 μL of supernatant was transferred to a new plate in which 100 μL of reaction mixture was added to each well, followed by incubation at room temperature in the dark for 30 min. The plate was read at 492 and 690 nm using a SpectraMax Plus 384 Plate Reader (Molecular Devices, Sunnyvale, CA, USA). The reference absorbance at 690 nm was subtracted from the absorbance at 492 nm. The readings from the untreated and test wells were subtracted from the reading from control wells containing medium alone. This yielded the corrected reading for the untreated or tests well. The percentage of cytotoxicity was calculated using the following formula:

(corrected reading from test well − corrected reading from untreated well)/
(reading from well with 100% dead cells in response to Triton X-100 − corrected reading from untreated well) × 100.

### Western blot analysis

Following 30 min of infection with *K. pneumoniae* (as described above), PMNs (4.5 × 10^6^ cells) were collected after 0, 2, 4, and 6 h and then re-suspended in 50 μL of RIPA buffer (Cell Signaling Technology, Danvers, MA, USA) supplemented with a protease inhibitor at −80°C overnight and centrifuged at 16,100 × *g* for 20 min at 4°C to obtain cell lysates. After discarding the cell debris, the supernatant protein concentrations were determined using the Bio-Rad Protein Assay Dye Reagent Concentrate (Hercules, CA, USA). A western blot analysis was performed and a housekeeping protein, COX-IV, was used as a control for equal loading. Samples were re-suspended in a loading buffer (50 mM Tris–HCl pH 6.8, 2% SDS, 0.1% bromophenol blue, 10% glycerol, 100 mM dithiothreitol), and heated for 5 min at 90°C. Denatured proteins (20–70 μg/sample) were separated on a 12% denaturing polyacrylamide gel by SDS-PAGE and transferred to nitrocellulose membranes (Hybond C; Amersham Biosciences, Little Chalfont, UK). Membranes were blocked for 1 h at room temperature with a 5% (w/v) non-fat dry milk solution containing 10 mM Tris–HCl pH 7.5, 140 mM NaCl, and 0.1% Tween 20 (TBS–T) before incubation overnight at 4°C with the primary antibodies anti-Bax, anti-BCL-_2_, anti-Mcl, anti-Caspase-3, or anti-COX-IV (all from Cell Signaling), anti-Bcl-xS/L, anti-FLIP (from Santa Cruz Biotechnology, Santa Cruz, CA, USA), anti-Caspase-8, and anti-X-chromosome-linked IAP (from BD Bioscience) diluted 1/100 to 1/1,000 with 1% BSA in TBS–T according to the manufacturers' instructions. After washing, the membranes were incubated for 1 h with a species-appropriate horseradish peroxidase-labeled secondary antibody (Santa Cruz Biotechnology) diluted 1/10,000 with 2.5% non-fat milk in TBS–T, and the labeled proteins were detected using Western Lightning Plus-ECL Detection Kit (Perkin–Elmer, Waltham, MA, USA). The signals were quantified by densitometry.

### DNA fragmentation assay

PMNs were infected with *K. pneumoniae* (as described above) or left untreated and DNA fragmentation was measured at various time points post-infection. Briefly, PMNs were incubated for 1 h at 37°C with TES lysis buffer [20 mM EDTA, 100 mM Tris, pH 8.0, 0.8% (w/v) SDS] containing 10 mg/mL RNase and then incubated overnight at 56°C with TES lysis buffer containing 20 mg/mL proteinase K for DNA preparation. The DNA was analyzed by electrophoresis on 1.6% agarose gels stained in Novel Green (GeneDireX, Atlanta, GA, USA) containing a TBE buffer for 30 min, following by de-staining in H_2_O. DNA ladders were visualized using a UV light source.

### Fas-stimulated apoptosis and caspase activity assays

PMNs were left untreated, infected with KP-M1 or DT-X (as described above), or treated with 500 ng/mL mouse anti-Fas IgM Ab (human, activating; Millipore, Temecula, CA, USA) in 5% pooled serum at 37°C for 30 min. The pellet was washed with HBSS, and the medium was then replaced with 5% pooled serum, followed by incubation at 37°C. PMNs caspase-3 activity was determined at various time points post-infection using ApoAlert Caspase Colorimetric Assay Kits (Clontech) according to the manufacturer's instructions. In each case, caspase-3 activity was assessed by quantifying the luminescence generated upon the cleavage of a caspase-specific proluminogenic substrate.

### Live/dead staining to visualize viable neutrophils

PMNs were infected with *K. pneumoniae* as described above. At various time points post-infection, PMNs were stained with a combination of SYTO-16 (Invitrogen, Carlsbad, CA, USA) and ethidium bromide (MDBio Inc., Taipei, Taiwan). Staining with 0.5 nmol SYTO-16 for 10 min at room temperature was used to visualize viable PMNs. This dye penetrates cell membranes and stains DNA. Consequently, the nuclei of viable cells appear green. Subsequent to SYTO-16 staining, the cells were stained with 0.5 nmol ethidium bromide for 5 min to reveal nonviable PMNs. Images were captured using an Axio Vision Digital Imaging System (Carl Zeiss Micro Imaging, Oberkochen, Germany).

### Cytokines measurements

PMNs were infected with *K. pneumoniae* (see description above), and culture supernatants were collected at various time points post-infection. Supernatants were stored at −80°C until use for cytokine determination. The IL-8, IL-10, and TNF-α concentrations were determined by enzyme-linked immunosorbent assays (ELISA) using commercial kits (R&D Systems, Wiesbaden-Nordenstadt, Germany). In some tests, 1 μg of anti-IL-8 blocking monoclonal antibody (R&D Systems, Minneapolis, MN, USA) or isotype control IgG (Sigma-Aldrich) was added to 20 μL of supernatant to estimate IL-8 depletion.

### Statistical analyses

Data obtained in experiments that included a control group and one experimental group were analyzed using unpaired Student's *t*-tests. Data obtained from studies containing multiple experimental groups were analyzed by one-way analysis of variance (ANOVA) followed by a Tukey's *post-hoc* tests. Comparative data obtained in the time course study are presented as normalized values with respect to the initial time of 1.0. Data were considered statistically significant when two-sided *p*-values were < 0.05.

## Results

### Neutrophils killed *K. pneumoniae* after infection

**Figure 1:**
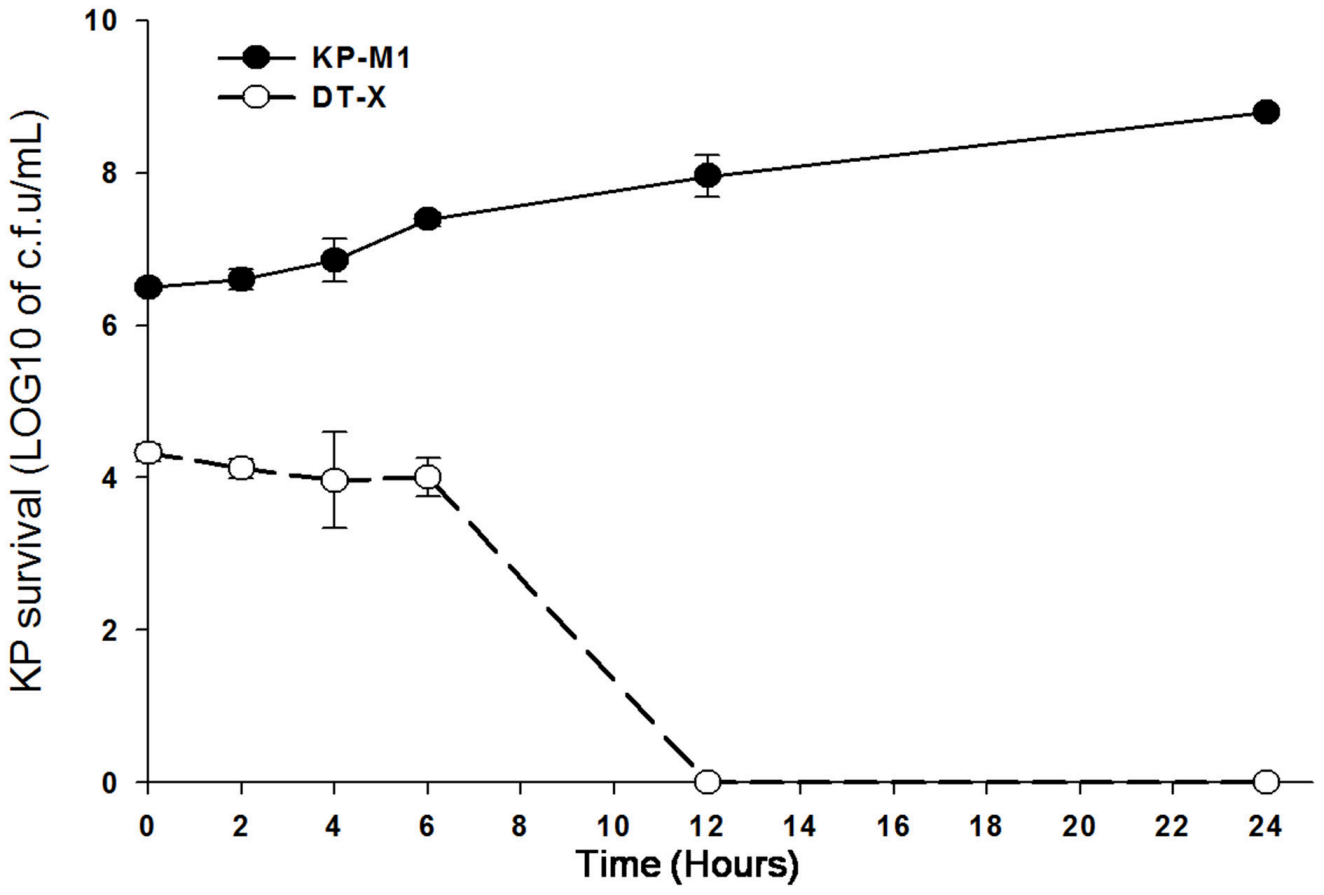
**The rate at which neutrophils from 5 healthy volunteers killed capsular (KP-M1) and acapsular (DT-X) ***K. pneumoniae*** serotype K1 after infection**. The counts of KP-M1 increased after infection, while the counts of DT-X declined to below the limit of detection after 12 h post-infection (MOI, 1:1). Evaluation at various times post *K. pneumoniae* infection of neutrophils. The first time point was as 30 min post infection of neutrophils. See text for details. It shows impaired KP-M1 clearance by neutrophils.

The viable bacterial counts (log_10_ cfu/mL) after neutrophils infected with *K. pneumoniae* for 30 min were 6.51 ± 0.43 for KP-M1 and 4.19 ± 0.37 for DT-X (Figure 1). The PMNs bactericidal activity against KP-M1 was significantly poor compared to the PMNs bactericidal activity against DT-X. After infection of PMNs, we withdraw the samples at 2, 4, 6, 12, 24 h post-infection to evaluate the colony counts. The viable counts of KP-M1 stabilized and increased after 6 h post-infection, whereas the viable counts of DT-X declined sharply to below the limit of detection after 12 h post-infection (Figure 1). It showed impaired KP-M1 clearance by PMNs.

### Cytotoxicity in neutrophils after infection with *K. pneumoniae*

**Figure 2:**
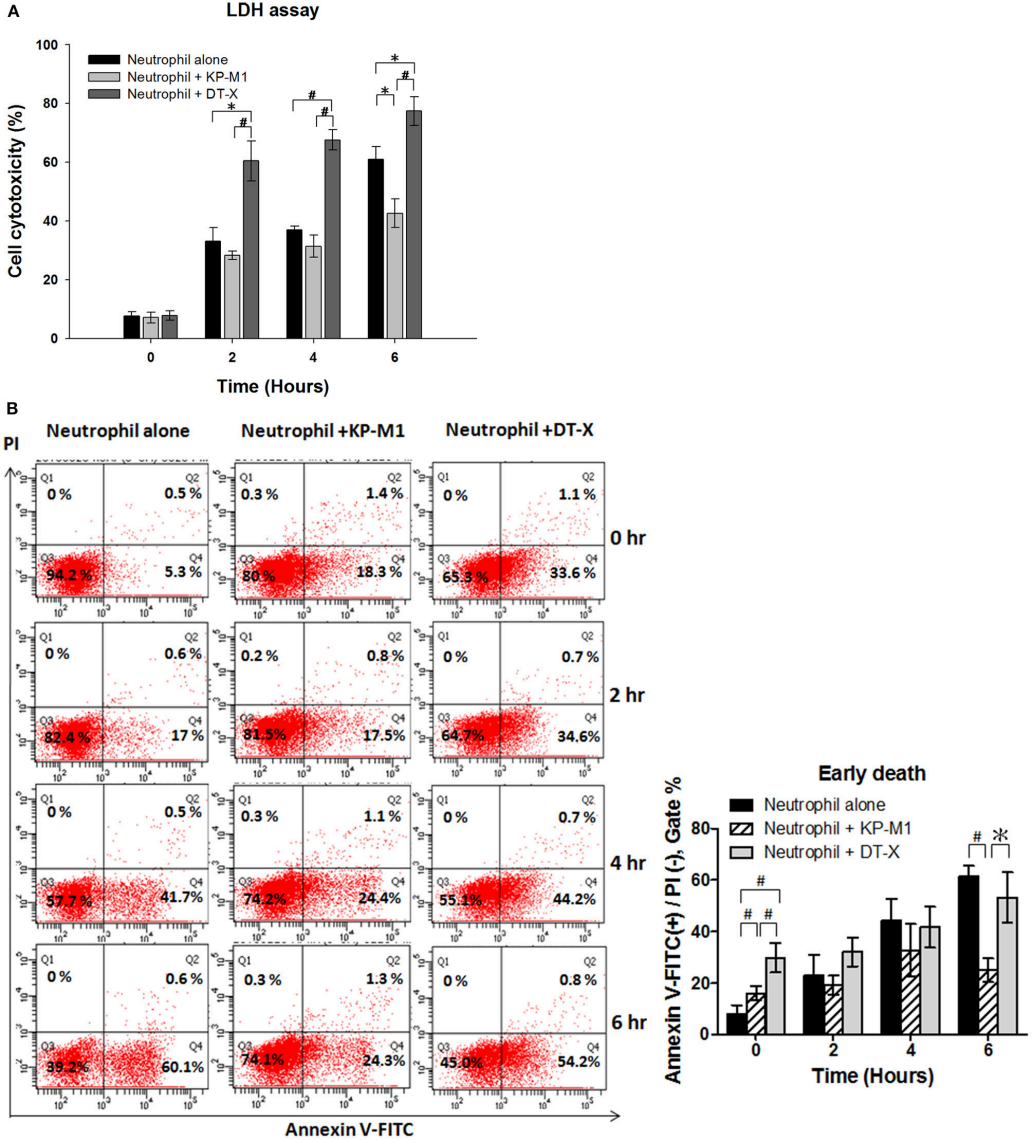
**Infection with capsular ***K. pneumoniae*** serotype K1 delays neutrophil apoptosis**. LDH release assay **(A)**. The percentage of cell cytotoxicity was calculated by measuring the amount of LDH released from the damaged cells into supernatant. The results show higher cell lysis on neutrophils infected with DT-X (MOI, 1:1). Cell cytotoxicity on PMNs infected with KP-M1 (MOI, 1:1) increased more slowly than that of PMNs infected with DT-X (MOI, 1:1) or on untreated PMNs (6 h post infection). Untreated cells and neutrophils infected with KP-M1 or DT-X were double-stained with annexin V-FITC and PI, then analyzed by flow cytometry **(B)**. Representative dot plot of untreated neutrophils and neutrophils infected with DT-X or KP-M1 (MOI, 1:1) at 0, 2, 4, and 6 h post-infection. Pooled flow cytometry data indicate the fraction of annexin V-positive but not PI–positive neutrophils at the indicated time points. Data are means ± SEM (*n* = 12). ^*^p < 0.05; ^#^*p* < 0.01.

LDH release assay showed increasing cell cytotoxicity on untreated PMNs in a time-dependent fashion (Figure 2A), results consistent with the induction of constitutive apoptosis in human PMNs over time (Figure 2B). In contrast, cell cytotoxicity on PMNs infected with KP-M1 increased more slowly than that of untreated PMNs or PMNs infected with DT-X (6 h post-infection; Figure 2A).

### *K. pneumoniae* infection caused neutrophils to undergo apoptosis

According to the flow cytometric diagram of annexin V-fluoresce in isothiocyanate (FITC)/ propidium iodide (PI) staining, this double-staining approach allows three subgroups of cells to be distinguished: viable, non-apoptotic cells are annexin V-negative and PI-negative (lower left quadrant); early apoptotic cells with intact membranes are annexin V-positive and PI-negative (lower right quadrant); and late apoptotic cells with compromised plasma membranes are annexin V-positive and PI-positive (upper right quadrant). Representative Fluorescence correlation spectroscopy (FCS) plots are shown in Figure 2B, and pooled data from 12 independent tests are also shown in Figure 2B. Our data demonstrate that PMNs infected with KP-M1 displayed a slow increase of PMNs with annexin V labeling but not more PI uptake than untreated PMNs, which are feature characteristics of delayed PMN apoptosis. PI staining of KP-M1-infected PMNs population was also increasing more slowly than untreated PMNs or DTX-infected PMNs, confirming that infection with KP-M1 maintained plasma membrane integrity of PMNs as indicated by the cell cytotoxicity data shown above.

### Apoptosis regulatory protein in human neutrophils following infection with *K. pneumoniae*

**Figure 3:**
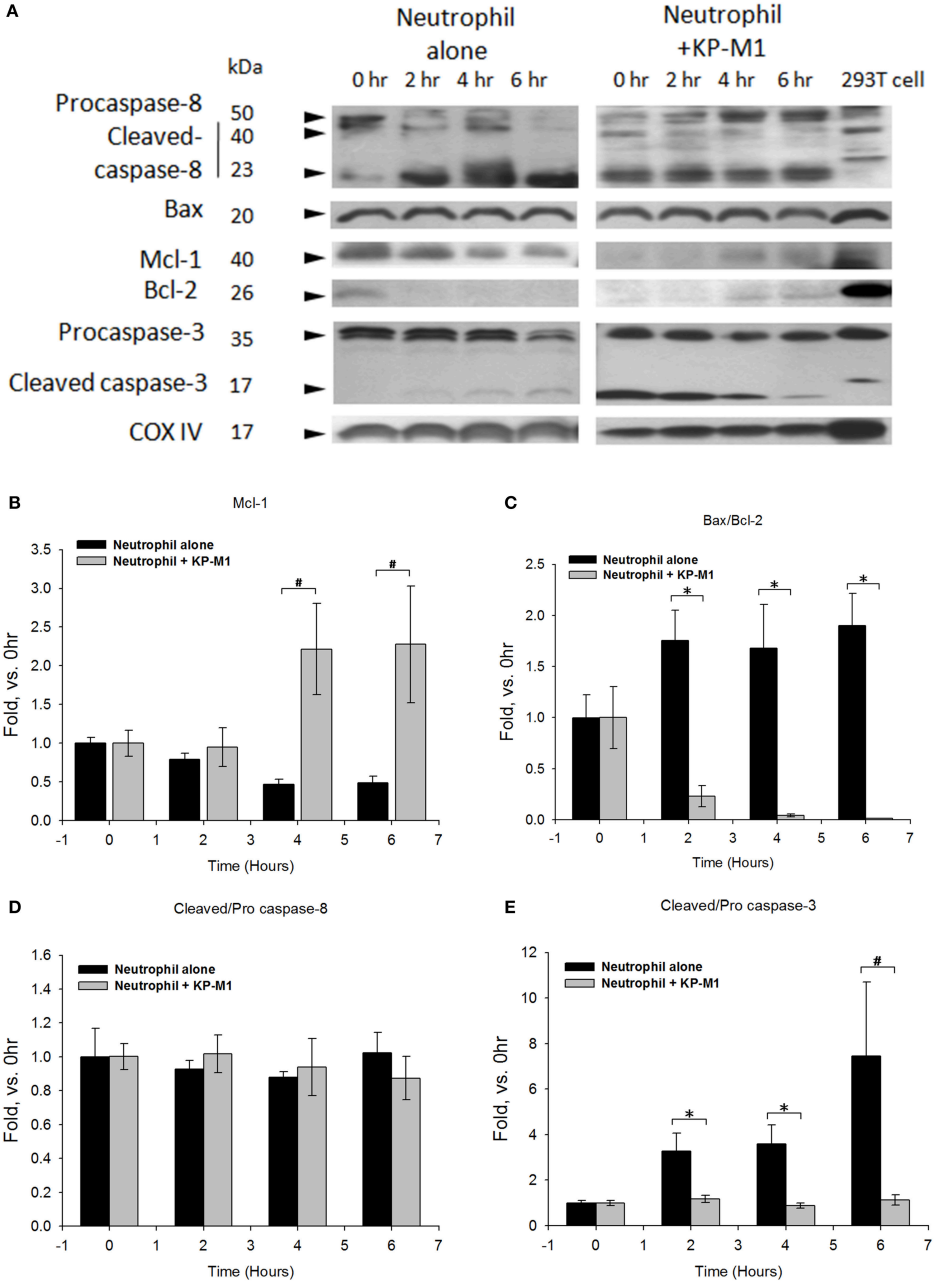
**Western blots for protein levels in human neutrophil following infection with capsular ***K. pneumoniae*** serotype K1**. Lysates from neutrophils that were in buffer alone, infected with KP-M1 (MOI, 1:1) as indicated were analyzed by immunoblot of molecules involved in DNA fragmentation at various time points post-infection. Shown is representative of six tests **(A)**. The Cox IV is used as internal loading control. To quantify immunoblot band intensity, densitometry analysis of Mcl-1, Bax to Bcl-2 ratio, cleaved caspase 8 to pro-caspase 8 ratio, and cleaved caspase 3 to pro-caspase 3 ratio were performed based on six separate tests. Data were plotted as the signal above untreated neutrophils vs. time 0 for indicated bands. Comparison of the immunoblot band intensity of untreated neutrophils, increasing amount of Mcl-1 **(B)**, decreasing expression of Bax to Bcl-2 ratio **(C)** resulted in less cleaved caspase 3 to pro-caspase 3 ratio **(E)** detected in KP-M1-infected neutrophils. However, no statistical difference in the ratio of cleaved caspase-8 to procaspase-8 **(D)** in untreated neutrophils and KP-M1-infected neutrophils was noted at all time points. Data are means ± SEM (*n* = 6). ^*^p < 0.05; ^#^*p* < 0.01.

Using Western blot techniques (Figure 3A) and quantified densitometry (Figures 3B–E), we followed the time course of apoptosis processing in untreated PMNs and KP-M1-infected PMNs. We found untreated PMNs were undergoing constitutive apoptosis; traces of the processing of procaspase-3 to its mature form were detected at 6 h post-infection. There is evidence that the Bax-to-Bcl-2 ratio appears to control the relative sensitivity or resistance of many cell types to apoptotic stimuli (Salakou et al., 2007). In comparison with the immunoblot band intensity of untreated PMNs, increasing amounts of Mcl-1 (Figure 3B) and decreasing expression ratio of Bax/Bcl-2 (Figure 3C), and less cleaved caspase-3/ procaspase-3 were detected in PMNs infected with KP-M1 in a time-dependent manner (Figure 3E). The FADD-like IL-1ß converting enzyme inhibitory protein (FLIP) is the main anti-apoptotic mechanism in the extrinsic pathway (Hotchkiss et al., 2009). It prevents activation of caspase-8 following ligation of Fas and the TNF-related apoptosis inducing lig and receptors and subsequent cleavage of effectors caspase. Another group comprises the family of inhibitors of apoptosis (IAPs) protein (Berends et al., 1994). We found no difference of FLIP and IAPs expression between untreated PMNs and KP-M1-infected PMNs (data not shown). As expected, there was no statistically significant difference in the ratio of cleaved caspase-8/procaspase-8 between untreated PMNs and KP-M1-infected PMNs at all time points post-infection (Figure 3D). These data demonstrate that, relative to untreated PMNs, activation of anti-apoptotic protein of Bcl-2, Mcl-1 was found in KP-M1-infected PMNs, suggesting that this organism acts on the Bcl-2 family protein in the apoptotic cascade to curtail activation of executioner caspase-3 and extend PMN lifespan. During the late stages of apoptosis, caspase-activated DNases cleaved nuclear DNA. The DNA fragmentation assay demonstrates that relative to untreated PMNs, DNA fragmentation of KP-M1 infected PMNs was significantly delayed at all time points examined between 12 and 24 h post-infection (see in Supplementary Material).

### Caspase-3 activity of neutrophils after infection with *K. pneumoniae*

**Figure 4:**
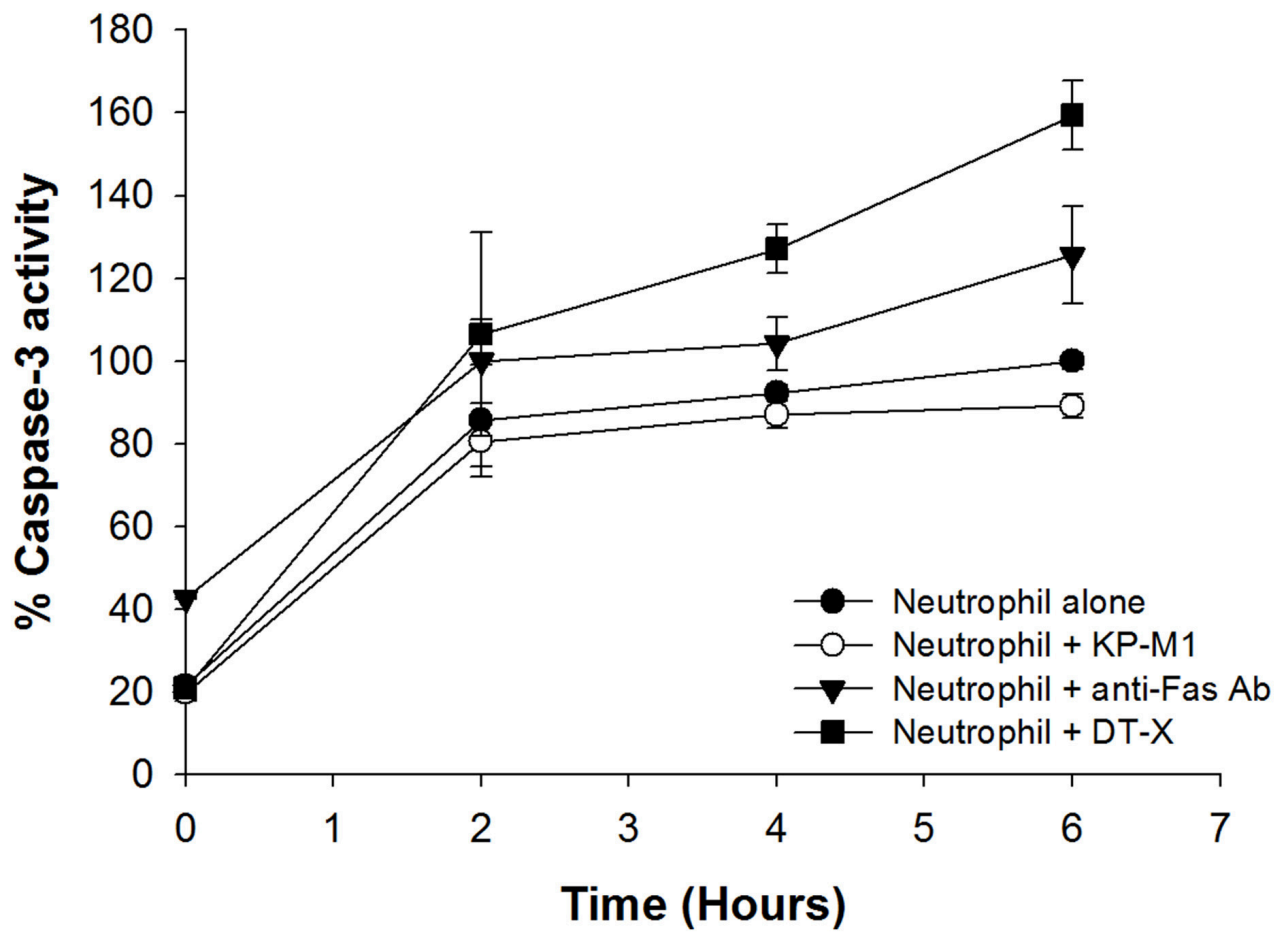
**Caspase-3 activity of neutrophil is impaired by capsular ***K. pneumoniae*** serotype K1**. Neutrophils were left untreated or were incubated with anti-Fas Ab (500 ng/mL), KP-M1 (MOI, 1:1), or DT-X (MOI, 1:1). Caspase-3 activity of neutrophil was assessed using the luminescence generated by a caspase-3-specific proluminogenic substrate at the indicted time points to quantify. The level of caspase activity for untreated neutrophils at 6 h is set as 100% (control), and all other data are expressed relative to the untreated neutrophils data point at 6 h. Data are means ± SEM (*n* = 12). Comparison of caspase-3 activity of untreated neutrophils or neutrophils infected with DT-X or neutrophils treated by anti-Fas Ab, KP-M1-infected neutrophils activated less caspase-3 activity (*p* < 0.05 at 2, 4, and 6 h post-infection).

Anti-Fas Ab promoted caspase-3 activation in PMNs was demonstrated as previously reported (Faouzi et al., 2001). Corresponding to the prompted increase in caspase-3 activity in PMNs infected with DT-X, PMNs treated by anti-Fas Ab, or untreated PMNs, KP-M1-infected PMNs activated less caspase-3 activity (*p* < 0.01 at 2, 4, and 6 h post-infection; Figure 4).

### Viability of human neutrophils following infection with *K. pneumoniae*

**Figure 5:**
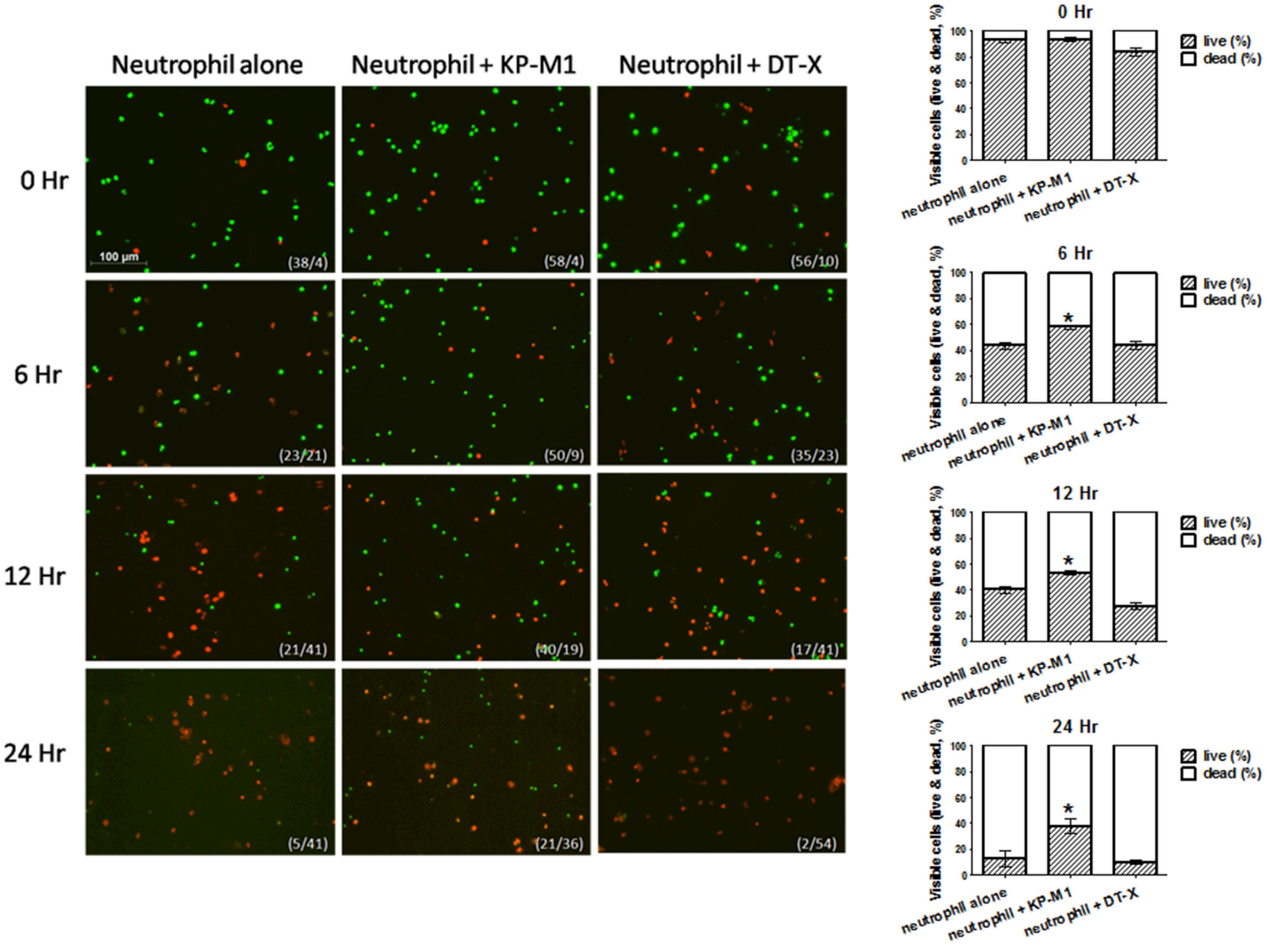
**Live/dead staining to visualize viable neutrophils after infection with ***K. pneumoniae*** serotype K1**. Live/dead staining was conducted on untreated neutrophils or neutrophils infected with KP-M1 or DT-X (MOI, 1:1) at 0, 6, 12, 24 h post-infections. The death (red staining) of neutrophils was increasing in a time-dependent manner. In comparison of the count of living cells of untreated neutrophils, or neutrophils infected with DT-X, more than 40% neutrophils infected with KP-M1 were living (green staining) after 24 h post-infection. The numbers in bottom right are live/dead cells. The right column shows live/dead cells at various time points post-infection (*n* = 10). Comparison of living cell counts of untreated neutrophils, KP-M1-infected neutrophils presents more living cell counts (^*^p < 0.05 at 6, 12, and 24 h post-infection).

A two-color immune-fluorescent staining (live/dead staining) was used to investigate PMNs survival after ingesting *K. pneumoniae* strains. After staining with SYTO 16 (green) and ethidium bromide (red), viable cells showed green staining, whereas dead cells with compromised membranes stained red. This staining demonstrated that death of PMNs increased in a time-dependent manner (Figure 5). The results showed that 24 h post-infection, more than 40% PMNs infected with KP-M1 were viable (green staining; Figure 5). In contrast, less than 10% untreated PMNs or those infected with KP-DT-X were viable (green staining; Figure 5).

### KP-M1 induced IL-8 reduces human neutrophil apoptosis

**Figure 6:**
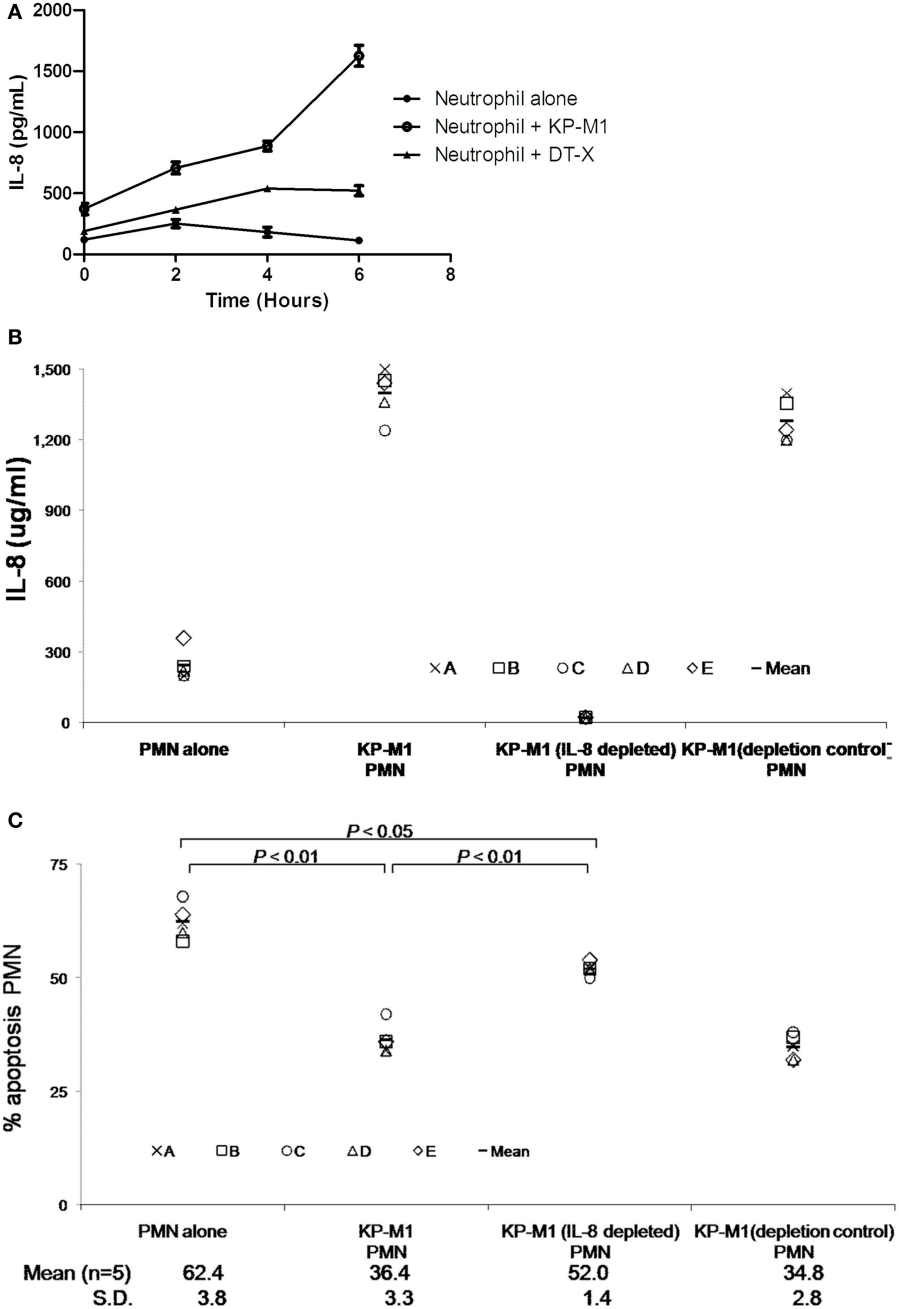
*****K. pneumoniae*** serotype K1 induces IL-8 reduces constitutive neutrophil apoptosis**. KP-M1 induces a time-dependent IL-8 release by neutrophils **(A)**. Neutrophils were left on untreated or infected with KP-M1 or DT-X (MOI, 1:1) and the IL-8 content of supernatants was measured at the given time points using ELISA. The data are the means ± SEM of duplicate assays for each condition obtained from three independent tests. The KP-M1 induces higher levels of IL-8 than DT-X. IL-8 depletion test **(B)**. Freshly isolated neutrophils from 5 healthy adults were co-incubated *in vitro* in medium alone, with KP-M1 (MOI, 1:1), with supernatant taken 6 h after neutrophils infected with KP-M1, and with these same supernatants depleted for IL-8 using a monoclonal anti-IL-8 serum or with a depletion control. The efficiency of IL-8 depletion was assessed using IL-8 ELISA. Untreated cells or neutrophils infected with KP-M1 (MOI, 1:1) were assessed for apoptosis after 6 h post-infection with or without IL-8 depletion **(C)**. The percentage of apoptotic neutrophils was determined by double-stained with annexin V-FITC and PI, then analyzed by flow cytometry. The data indicate the fraction of annexin V-FITC positive but no PI positive neutrophils at different conditions. It demonstrates that depletion of IL-8 from supernatant restores the neutrophils constitutive apoptosis, even neutrophils infected with KP-M1.

Chemokine production by PMNs is thought to affect the inflammatory process by recruiting various activated leukocyte populations. As the primary target cells of IL-8 are PMNs, the production of this chemokine by inflammatory PMNs appears to serve as an amplification loop, attracting more PMNs to the site of inflammation. IL-8 was measured in the supernatants of PMN cultures with or without infection with KP-M1 or DT-X at various time points post-infection. It showed KP-M1 or DT-X infected PMNs produced IL-8 in a time-dependent fashion and KP-M1 induces higher levels of IL-8 than DT-X (Figure 6A). KP-M1 infected PMNs did not induce the production of IL-10 and TNF-_α_ (data not shown). IL-8 has been reported to inhibit the constitutive apoptosis of PMNs (Dunican et al., 2000). To investigate whether IL-8 was responsible for anti-apoptotic effect of the supernatants, IL-8 was depleted from the supernatants taken from PMNs-KP-M1 co-cultures at 6 h after infection. Using immunoprecipitation with anti-IL-8 or isotype control IgG, the IL-8 concentration in the supernatants was reduced from 1398 ± 101.8 to 22.4 ± 2.2 μg/mL (*n* = 5; Figure 6B). Co-incubation of these samples with PMNs demonstrated that depletion of IL-8 from the supernatant restored the KP-M1 infected PMNs constitutive apoptosis (+Annexin V/-PI flow cytometry) from 36.4 ± 3.3 to 52.0 ± 1.4% (*p* < 0.01; Figure 6C).

## Discussion

Serotype K1 is the main serotype culprit in *K. pneumoniae*-mediated invasive syndrome (Fang et al., 2007). Therefore, it is of interest to understand how the K1 virulence factor, particularly the capsule, contributes to the pathogenesis of a distinctive invasive syndrome. Although PMNs participate in the first line of defense against bacterial disease, they may no longer play a defensive role when specific bacterial factors are involved. A variety of ways for the pathogens to take over and control the host cells have been found in different pathogens (Weinrauch and Zychlinsky, 1999). In this study, we contend that PMNs can serve as host cells for *K. pneumoniae* serotype K1. This culprit was not destroyed; instead, the ingested bacteria survived within PMNs.

Although untreated PMNs became apoptotic within 10 h, 40% of infected PMNs could survived up to 24 h post-infection in this study; PMNs infected with *K. pneumoniae* serotype K1 significantly delayed the constitutive apoptosis of PMNs, detected by morphological analysis, annexin V, DNA fragmentation assay, and Western blot analysis. The observed anti-apoptotic effect was associated with a markedly lower level of caspase-3 activity in KP-M1-infected PMNs. The sequential activation of caspases is the last stage of cellular destruction and denotes the apoptosis of the host cells (Hotchkiss et al., 2009). There have been several pathogens found to be capable of changing the threshold of apoptosis in PMNs, the issue being that to avoid PMNs-mediated destruction they activate apoptosis (Watson et al., 1996; Rotstein et al., 2000). In contrast, we found that *K. pneumoniae* serotype K1 affected PMN constitutive apoptosis, which resulted in delayed apoptosis in the early stage of infection. The *K. pneumoniae* serotype K1 can extend the lifespan of PMNs, making them suitable host cells for bacterial survival and multiplication.

The two main pathways of apoptosis induction in PMNs are intrinsic or extrinsic, and both culminate in the activation of caspases. The extrinsic pathway involves death receptors (e.g., Fas) via specific ligands; the intrinsic pathway can be started by several factors, such as cellular stress including DNA damage or growth factor deprivation (Hotchkiss et al., 2009). One issue surrounding the study of PMNs is that they have a relatively short lifespan if found in peripheral blood, and enter apoptosis after 6–10 h (Cabrini et al., 2010). We found that KP-M1 could extend cell lifespan of PMNs to more than 24 h post-infection (Figure 5). Compared with untreated PMNs or PMNs infected with DT-X, or PMNs treated by anti-Fas Ab, we found that KP-M1-infected PMNs activated less caspase-3 activity (Figure 4). The three groups of anti-apoptotic genes that counteract caspase activation through extrinsic and intrinsic apoptotic pathways are FLIP, Bcl2 family such as Mcl-1, and IAP (Busca et al., 2000). The pro-apoptotic mechanisms by bacteria include the activation of several pro-apoptotic proteins (e.g., caspases), the inactivation of anti-apoptotic proteins, and the up-regulation of endogenous receptor/ligand systems (Grassmé et al., 2001). The change of the ratio of Bax to Bcl-2 can stimulate the release of cytochrome c, which activates caspase-9 and caspase-3, and it may govern sensitivity to apoptotic stimuli (Bartchewsky et al., 2010). We found no difference of FLIP and XIAP expression between untreated PMNs and KP-M1-infected PMNs. Time-dependent modulations of the ratios of Bax to Bcl-2 and MCl-1 in PMNs infected with KP-M1 were found. It is tempting to speculate that the anti-apoptotic activity exerted by the bacteria during the early stages of infection helps maintain both the integrity and the metabolic activities of the infected cells to allow bacterial proliferation. *Mycobacterium tuberculosis* is typically used in investigating host-mediated macrophage apoptosis, as it seems to lengthen intracellular replication by curtailing apoptosis (Marriott et al., 2005). There is an imbalance among the Bcl-2 family of proteins, with regards to its anti-apoptotic members, which frequently occurs in cancer cells and has been linked to tumor cell survival and apoptosis resistance (Ola et al., 2011). Our data showed KP-M1 delayed PMNs cell death, but further study is needed to confirm that KP-M1 is specifically causing inhibition of the intrinsic pathway of apoptosis. The modulation of the pro- and anti-apoptotic effects of PMNs infected with KP-M1 at different stages of infection may be controlled and determined by the signals encountered by the extracellular milieu, such as cytokines. It would be interesting to examine what different roles cytokines may play in the pathogenesis of KP-M1 in modulating the pro- and anti-apoptotic activities. In contrast to the response to untreated PMNs, KP-M1-infected PMNs produced significantly more IL-8 in a timely manner. The IL-8 was shown to delay the constitutive apoptosis of PMNs (Kettritz et al., 1998). In our study, PMNs produces a high amount of IL-8 upon co-culture with *K. pneumoniae* serotype K1, especially at later time points (Figure 6A). Subsequently, we also found that the IL-8-containing supernatants had a strong anti-apoptotic effect on freshly isolated PMNs. Depletion of IL-8 from the supernatants of *K. pneumoniae* serotype K1 and PMN co-cultures significantly decreased the anti-apoptotic activity of these supernatants. These data might indicate that the anti-apoptotic effect of *K. pneumoniae* serotype K1 is at least partially mediated by the autocrine production of IL-8 by PMNs.

Bacteria are known to trigger phagocytosis-induced apoptosis (Watson et al., 1996), but at low bacteria to neutrophil ratios (MOI, 1:1), acapsular Gram-negative bacteria could delay constitutive neutrophil apoptosis (Watson et al., 1996). This might be due partly to the lipopolysaccharide in these Gram-negative bacteria (Colotta et al., 1992). It is reported that LPS acts on Toll-like receptor 4 and for consequence of this binding, occurs activation and increased lifetime of the cells. A role for NF-κB, and also MAPKs, including p38 and extra cellular signal—regulated kinase, effects of LPS on neutrophil apoptosis, the transcriptional activity of Mcl-1, Bcl-XL, and Bax apoptosis-associated factors do not appear to play a major role in this process (Sabroe et al., 2005). Recently, a study conducted by Kobayashi et al. (2016) showed carbapenem-resistant ST258 *K. pneumoniae* strains enhance the survival of neutrophils after phagocytosis. The ST258 *K. pneumoniae* survival is mostly due to the lack of phagocytosis instead of being due to an intracellular persistence. They also found this ST258 *K. pneumoniae* strains delay neutrophil apoptosis and neutrophil was viable following 6 h exposure to these organisms. Another work found that the ST23 serotype K1 *K. pneumoniae's* escape from neutrophil intracellular killing results in dissemination (Lin et al., 2010). The interaction of neutrophils with different ST types of *K. pneumoniae* seems not to be the same. Our results provide an additional dimension to the ability of ST23 serotype K1 *K. pneumoniae*, most causative of the invasive syndrome, to alter innate host response by regulating neutrophil apoptosis.

In summary, we report that human PMNs undergo apoptosis after encountering live *K. pneumoniae* serotype K1 through a mechanism that is associated with modulation of the pro- and anti-apoptotic effects, which delays caspase-3 activation in PMNs and is accompanied by inducing the anti-apoptotic cytokine IL-8. This suggests that *K. pneumoniae* serotypes K1 can extend the lifespan of PMNs, making them suitable host cells for bacterial survival and multiplication after infection occurs. This organism may use PMNs as “Trojan horses” for subsequent infection of other cells. This effect may contribute to the development of a distinctive invasive syndrome.

## Author contributions

CL, SC, and WT designed the study and interpreted data; CL and SC contributed to manuscript drafting. CC and FC performed experiment; CL and CC performed the statistical analysis. All authors read and approved the final manuscript.

### Conflict of interest statement

The authors declare that the research was conducted in the absence of any commercial or financial relationships that could be construed as a potential conflict of interest.
