# Supplementary figures and images for: Delay in Human Neutrophil Constitutive Apoptosis after Infection with Klebsiella pneumoniae Serotype K1
Source: Front Cell Infect Microbiol. 2017 Mar 27;7:87. doi: 10.3389/fcimb.2017.00087 (PMC5366327; doi:10.3389/fcimb.2017.00087)

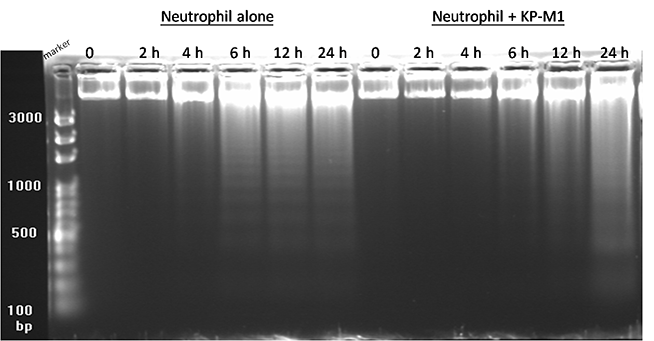

Supplement: Supplementary file 1 [file Image1.TIF]
